# The Use of Hot Melt Extrusion to Prepare a Solid Dispersion of Ibuprofen in a Polymer Matrix

**DOI:** 10.3390/polym15132912

**Published:** 2023-06-30

**Authors:** Kinga Biedrzycka, Agnieszka Marcinkowska

**Affiliations:** 1 Institute of Chemical Technology and Engineering, Poznan University of Technology, Berdychowo 4, 60-965 Poznan, Poland; 2 Applied Manufacturing Science Sp. z o.o, Krzemowa 1, 62-002 Złotniki, Poland

**Keywords:** hot melt extrusion, Eudragit, ibuprofen, amorphous solid dispersion

## Abstract

In this work, we report the use of the hot melt extrusion method in harsh extrusion conditions, i.e., screw rotation speed of 250 rpm, temperature above 100 °C, and two mixing zones, in order to obtain an amorphous dispersion of an active pharmaceutical ingredient (API) that is sparingly soluble in water. As a polymer matrix Eudragit EPO (E-EPO) and as an API ibuprofen (IBU) were used in the research. In addition, the plasticizer Compritol 888 ATO (COM) was tested as a factor potentially improving processing parameters and modifying the IBU release profile. In studies, 25% by weight of IBU, 10% of COM and various extrusion temperatures, i.e., 90, 100, 120, 130, and 140 °C, were used. Hot melt extrusion (HME) temperatures were selected based on the glass transition temperature of the polymer matrix (T_g_ = 42 °C) and the melting points of IBU (T_m_ = 76 °C) and COM (T_m_ = 73 °C), which were tested by differential scanning calorimetry (DSC). The thermal stability of the tested compounds, determined on the basis of measurements carried out by thermogravimetric analysis (TGA), was also taken into account. HME resulted in amorphous E-EPO/IBU solid dispersions and solid dispersions containing a partially crystalline plasticizer in the case of E-EPO/IBU/COM extrudates. Interactions between the components of the extrudate were also studied using infrared spectroscopy (FTIR-ATR). The occurrence of such interactions in the studied system, which improve the stability of the obtained solid polymer dispersions, was confirmed. On the basis of DSC thermograms and XRPD diffractograms, it was found that amorphous solid dispersions were obtained. In addition, their stability was confirmed in accelerated conditions (40 °C, 75% RH) for 28 days and 3 months. The release profiles of prepared tablets showed the release of 40% to 63% of IBU from the tablets within 180 min in artificial gastric juice solution, with the best results obtained for tablets with E-EPO/IBU extrudate prepared at a processing temperature of 140 °C.

## 1. Introduction

The problem faced by the pharmacy today is drugs that are insoluble in water [1]. Obtaining a drug form that is assimilable by the patient’s body is therefore a challenge. One of the methods to improve this parameter is to obtain an amorphous solid dispersion or a solid solution of active pharmaceutical ingredient (API) in a polymer matrix [2]. The amorphic form of the drug is better absorbed by the body than its crystalline form [3]. However, such an amorphous form of API is thermodynamically unfavorable, therefore recrystallization processes can occur during storage. To prevent this, systems with appropriate glass transition temperature are obtained in which intermolecular interactions occur between the ingredients like hydrogen-bonding interactions, ionic interaction, or other non-specific interactions [3,4,5,6]. Ibuprofen (IBU) can therefore be incorporated into a matrix of methacrylic polymers as there are intermolecular interactions between these ingredients [7].

There are several methods used in the pharmaceutical industry to obtain amorphous solid dispersions, such as coprecipitation, spray-drying [8,9], cryomilling [10], and hot-melt extrusion [2,11]. Hot melt extrusion method (HME), which has been developing in pharmacy since the 1970s [5,6,7,8,9], has many advantages over solvent methods, i.e., no use of solvent, a wide range of dosage forms and delivery routes, continuous operation, and fewer processing steps [2]. However, due to the process being carried out at elevated temperature, high shear force, and pressure, degradation of the polymer and sensitive API may occur. Shear stress is related to the mechanical energy generated by the kneading elements of the extruder, which must exert the appropriate energy to convert the polymer-crystalline powder system into the desired form of amorphous dispersion [2,11]. The temperature of the process, and thus the possibility of degradation of the ingredients, can be lowered by introducing a plasticizer that lowers the glass transition temperature of the polymer, as well as facilitates its processability. However, too high content of plasticizer can make the material too flexible, which will make further processing (e.g., milling) difficult. Some APIs can also exert a plasticizing effect on the polymer matrix, for instance ibuprofen [2] used in the present investigations.

The HME method can be used to obtain solid IBU dispersions in matrices of methacrylic polymers, such as Eudragit [12]. In one of the papers, the influence of process parameters on the properties of IBU—Eudragit L100-55 extrudates was investigated [2]. Authors used 25% and 33% of IBU and obtained amorphous solid dispersion by HME with 100 rpm and 150 rmp screw rotating speed and temperature above 100 °C (120 °C and 140 °C). Solid dispersion was more stable with lower amount of API in polymer matrix. Another study investigated the effect of polymer matrix modification on the properties of the obtained extrudates. Eudragit EPO was used as a reference matrix, and a number of methacrylic copolymers were polymerized. Structural modifications increased the mechanical stability of the tablets by changing the glass transition temperature of the polymer, while not affecting the drug release characteristics and the properties of forming amorphous solid solutions [11]. In other works, the IBU system—Eudragit EPO was also studied, but without the drug release process from the tablets [13] or using a small addition of IBU, i.e., 10% [14,15]. The stability of amorphous solid dispersion can be improved by using rather mild HME processing conditions, i.e., temperature below T_m_ of IBU, and small screw rotation speed (60 rpm) [13]. When higher rotation speed during HME process was used, i.e., 200 rpm, and only 10% of IBU, for stabilization of ASD intermolecular interactions are required to prevent recrystallization [14]. Recrystallization of IBU can also take place during the dissolution test, which is undesirable, as it reduces the concentration of the API in solution and API bioavailability [15]. Another Eudragit-type RSPO was used for transdermal coatings preparation [16] or tablets, but using a single screw extruder to obtain Eudragit EPO ibuprofen extrudates additionally containing microcrystalline cellulose [17]. Also, other types of polymers, like PVA [18], polyethylene oxide [19], polyethylene glycol 6000 (PEG 6000) with glyceryl monostearate [20], or sucroesters, which are obtained by esterifying sucrose with edible fatty acids [21], were used as a matrix for IBU incorporation by HME.

In this work, we examined the effect of the processing temperature and the addition of a plasticizer on obtaining a solid dispersion of ibuprofen in Eudragit EPO and the process of releasing the active substance in a simulated gastric juice. A significant addition of the active substance, 25% by mass, and harsh processing conditions (high-speed screw rotation, i.e., 250 rpm, two mixing zones, high temperature 90–140 °C for short residual time), was used in the work, which may affect the stability of the amorphous solid dispersion. In earlier studies conducted under the direction of Y. Tian [13], the temperature of processing was below the melting point of ibuprofen and slightly higher than the T_g_ of the polymer, hence mild processing conditions were used, which may affect the long residence time of the mixture in the extruder. In this work, processing temperatures above the melting point of ibuprofen and more stringent processing conditions were used. In addition, stability tests were conducted for 3 months for tablets. The processing of the extrudates into tablets causes additional stresses on this material during mechanical processing, which may affect the stability of the amorphous solid dispersion. What is more, the addition of Compritol was used as a lubricant, facilitating processing, which additionally, due to the lipid structure, may affect the process of releasing the active substance. The obtained extrudates were characterized by thermal methods, i.e., DSC and TGA to determine phase transitions and thermal stability of materials. Additionally, intermolecular interactions using infrared spectroscopy were studied, as they affect the stability of the extrudate.

## 2. Materials and Methods

### 2.1. Materials

**Figure 1:**
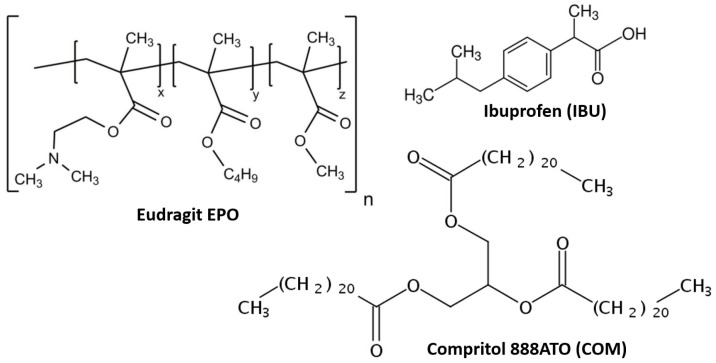
Materials used for investigations: Eudragit EPO, ibuprofen, Compritol 888ATO.

HME process. Polymer: Eudragit EPO, i.e., methacrylic acid copolymer in which the molar ratio of dimethylaminoethyl methacrylate: butyl methacrylate: methyl methacrylate is 2:1:1 (Evonik Rohm, Darmstadt, Germany), active pharmaceutical ingredient: Ibuprofen, i.e., (RS)-2-[4-(2-methylpropyl)phenyl]propionic acid (IBU, Shandong Xinhua Pharmaceutical I&E Co, Zibo, China), and Compritol 888ATO, i.e., glycerol dibehenate (COM, Gattefossé, Saint-Priest, France). The structures of the materials used for testing are shown in Figure 1.

Tablets preparation: lactose monohydrate, corn starch, calcium hydrogen phosphate dihydrate, microcrystalline cellulose, sodium carboxymethyl starch, colloidal silica, and magnesium stearate were purchased from JRS Pharma, Rosenberg, Germany.

For the release of API, an artificial gastric juice was prepared with sodium chloride, pepsin, hydrochloric acid (Merck KGaA, Darmstadt, Germany).

### 2.2. Methods

#### 2.2.1. Hot Melt Extrusion

**Figure 2:**
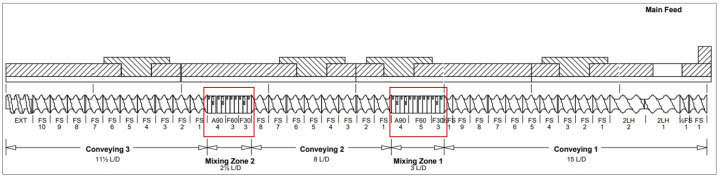
Screw system with two mixing zones.

Physical mixtures of Eudragit EPO with ibuprofen containing 25 wt% of API, and containing additionally 10 wt% of Compritol 888 ATO, were weighed, sieved through a 0.8 mm mesh sieve, and then mixed in a mixer. After that, prepared mixtures were processed using Pharma 11 counter-rotating laboratory scale twin-screw extruder with two mixing zones (Figure 2).

The temperature of the extruder die was set to 90, 100, 120, or 140 °C, and a used temperature profile of the barrel is presented in Table 1. Variable feed rates of the prepared mixture of ingredients to the extruder were used, i.e., 2–300 g∙h^−1^, with the samples taken at the feed values: 25 g∙h^−1^. The rotation speed of the screws was fixed at 250 rpm. The mixtures were extruded through a circular die (diameter 3 mm), then extrudates were cooled on a conveyor belt at room temperature and cut into granular samples. Therefore, the physical blend was manually fed into the extruder, and the solid dispersions of ibuprofen were collected on a conveyor belt, air-cooled, and stored in screw-capped glass bottles protected with parafilm against moisture at room temperature.

#### 2.2.2. Milling of Extrudates

The extrudates were milled in Quadro Comil sieve size 1.0 mm at a rotor speed of 275 rpm. The obtained powdered polymer-IBU or polymer-IBU-COM dispersions were stored in a desiccator over a drying agent.

#### 2.2.3. Tablets Preparation

Weighed components, presented in Table 2, for individual systems were mixed in a mixer Erweka AR401 for a period of 20 min at 17 rpm, and finally, magnesium stearate was added and mixing was continued for 5 min at the same speed, i.e., 17 rpm. After that, the mixture of ingredients was put into a rotary tablet press Korsch XL10 and compressed into round, biconvex tablets. The main compression was equal to 9.4–9.6 kN.

#### 2.2.4. Dissolution Tests

The dissolution tests were performed in artificial gastric juice (pH 3.0) solution as well and in a 20 mL vessel, at a temperature of 37 °C and a stirring speed of 100 rpm. These parameters are designed to simulate gastric conditions. For the preparation of artificial gastric juice, 2.0 g of sodium chloride and 3.2 g of pepsin powder were dissolved in 1000 mL of water. Then, 80 mL of hydrochloric acid (1 mol∙L^−1^) was added and made up to 1000.0 mL with water. A standard calibration curve of known amounts of IBU was used to quantify the amount of IBU released using a UV spectrophotometer at a wavelength of 264 nm.

The assessment of changes in concentration during the study of IBU released from tablets in prepared solutions was evaluated using the reverse-phase high-performance liquid chromatography method coupled with a diode array detector, RP-HPLC-DAD. The prepared tablet of known weight (containing 50 mg of API) was placed in the vessel. The medium was maintained at 37 °C and agitated at 50 rpm. The samples for testing the concentration of IBU in the acceptor fluid were collected at specific time intervals (from 0 to 180 min). The collected volumes of the acceptor fluid solution were supplemented with the appropriate amount of the acceptor fluid at the same temperature and containing no substance. The collected samples were filtered through a syringe filter with a diameter of 0.45 µm. IBU concentration was determined using the HPLC-DAD method.

The concentration of ibuprofen was determined by Agilent 708-DS Dissolution Apparatus (Agilent, Santa Clara, CA, USA) equipped with a UV detector at 264 nm. The column was Kinetex C18 (5 µm, 100 × 2.10 mm), and the mobile phase was composed of 0.1% formic acid and acetonitrile solution (60:40, *v/v*). A flow rate 0.7 mL∙min^−1^ were employed.

#### 2.2.5. Fourier Transform Infrared Spectroscopy (FTIR-ATR)

FTIR was used to identify the interactions between the polymer matrix and IBU, or between the polymer matrix, IBU, and COM. Infrared spectra were collected using a Nexus Nicolet 5700 Fourier Transform Infrared Spectrophotometer (FTIR, Thermo Electron Scientific Instruments Corporation, Madison, WI, USA) equipped with an attenuated total reflection (ATR) accessory with a diamond crystal at room temperature in range 4000–600 cm^−1^ and resolution 4 cm^−1^ at 64 scans.

#### 2.2.6. Differential Scanning Calorimetry (DSC)

The thermal properties of the pure ingredients and extrudates were analyzed using a DSC1 instrument (Mettler-Toledo, Greifensee, Switzerland). The sample (3–5 mg) was cooled with an intracooler to −50 °C and heated to 140 °C with a rate of 10 °C∙min^−1^. The glass transition temperatures (T_g,_ taken as a midpoint) and melting temperatures (T_m_) were determined from the heating cycles in the DSC traces, and were estimated using the automated tools in the DSC1 software STAR^e^.

#### 2.2.7. Thermogravimetric Analysis (TGA)

Thermogravimetric analysis (TGA) was performed using a Tarsus analyzer instrument TG 209 F3 (NETZSCH- Geratebau GmbH, Germany) to evaluate the thermal stability and composition of the samples. Samples in the form of powder weighing about 20 mg were placed in platinum crucibles and then on the thermobalance support. The measurement was carried out with a heating rate 10 °C∙min^−1^ in the temperature range of 30–600 °C under a nitrogen atmosphere (protective gas flow of 10 mL∙min^−1^ and purge gas 20 mL∙min^−1^).

#### 2.2.8. X-ray Powder Diffraction

XRPD was used to characterize the solid-state properties of the pure components and extrudates using an X-ray diffractometer TUR-M 62 used in the X-ray examinations. The following measurement parameters were used: lamp with Cu anode as a radiation source—wavelength Kα = 1.5418 Å, cathode supply voltage: 30 kV, lamp (current: 25 mA), range of 2θ diffraction angles: 5–30° or 10–30°, pulse counting step: 0.04°/2θ = 3 s.

## 3. Results and Discussion

### 3.1. Physical Characterization of Extrudates

**Figure 3:**
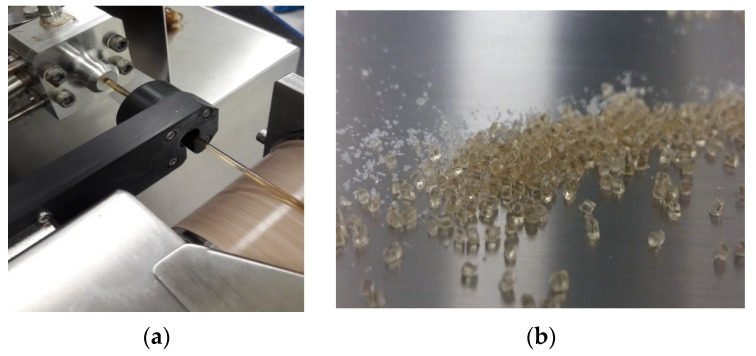
The polymer-IBU system (**a**) during the extrusion process and (**b**) after pelletization.

During extrusion, attention was paid to the color of the emerging extrudate, and in the entire range of applied temperatures it was close to straw-yellow (Figure 3). Moreover, the obtained Euragit-IBU extrudates were transparent, while those with the addition of COM plasticizer were opaque. This is due to the partial insolubility of the lipophilic plasticizer in the Eudragit EPO—ibuprofen system.

#### 3.1.1. Thermal Properties

In the first stage of the research, the characteristics of the ingredients were carried out. The temperatures of the characteristic phase transitions, i.e., the glass transition temperature of the polymer and the melting points of the API and the plasticizer, were determined. These temperatures are important for determining processing parameters because the extrusion is carried out above the glass transition temperature of the polymer, which leads to a reduction of its viscosity and promotes uniform flow during processing. Eudragit EPO glass transition occurs at a fairly low temperature, i.e., 42 °C, which is consistent with the literature [11]. On the other hand, IBU and COM melt at similar temperatures, i.e., T_m_^IBU^ = 76 °C (also confirmed in the literature [2]) and T_m_^COM^ = 73 °C (similarly to the literature [22]). In addition, the extrusion process should be conducted below the degradation temperature to avoid degradation of the ingredients, which would result in product contamination. Thermal stability tests were carried out using the TGA method. As can be seen from the results shown in Table 3, ibuprofen degrades at the lowest temperature, i.e., 126 °C; however, the matrix is more temperature-stable and its degradation starts above 200 °C. Based on the obtained results, a mixture of Eudragit/IBU was extruded and its thermal stability is higher than that of pure IBU. Hence, the extrusion temperature range was set at 90–140 °C.

**Figure 4:**
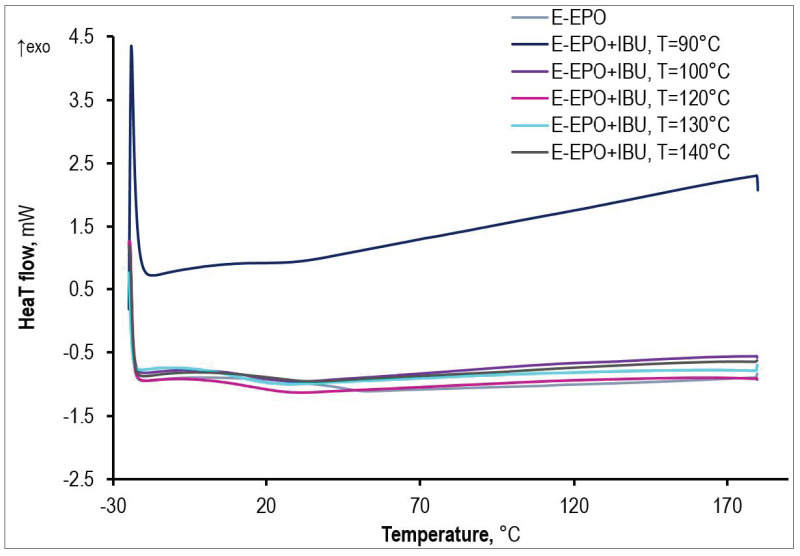
DSC thermograms of extrudates matrix/drug and pure matrix obtained at different processing temperatures.

Extrudates containing Eudragit EPO and ibuprofen have only one T_g_, which appears around 15 °C (Figure 4). Thus, ibuprofen exerts a plasticizing effect on Eudragit EPO, lowering the T_g_ of the pure polymer by about 30 °C. In addition, there is no melting peak of ibuprofen on the thermograms, which proves that the drug is completely dissolved in the matrix; it is in an amorphous form, and therefore it is in a form with better bioavailability. Thus, solid polymer-API dispersions were obtained in all cases.

**Figure 5:**
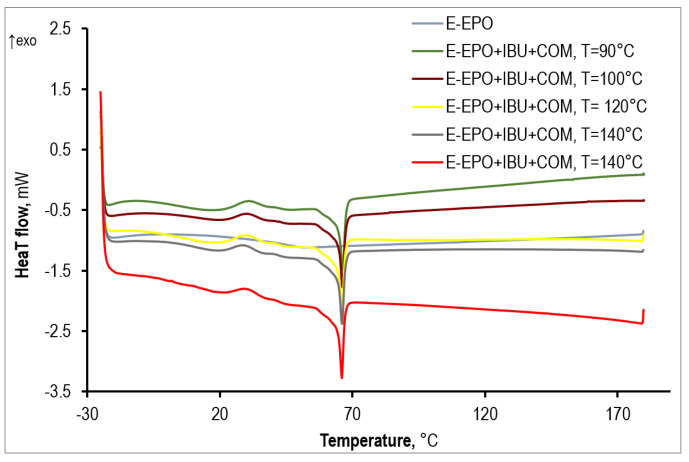
DSC thermograms of extrudates matrix/drug/plasticizer and pure matrix obtained at different processing temperatures.

In contrast, materials consisting of Eudragit EPO, ibuprofen, and COM 888 ATO show two T_g_ (Figure 5). The first T_g_ occurs around 10 °C and the second T_g_ around 45 °C. In addition, the thermograms show a melting peak at a temperature of about 66 °C, which indicates a partial content of the plasticizer COM in the crystalline form. Thus, the addition of COM to the polymer/IBU system causes a further decrease of the T_g_ (to the value of about 5–10 °C), which proves at least partial dissolution of the plasticizer in the matrix. The second T_g_ determined from the thermograms is, however, very close to the T_g_ of the pure matrix. This suggests the existence of more phases in the material when COM is added to the system. One phase is then probably a mixture of polymer, drug, and plasticizer, and the other is the pure polymer matrix. There is also a third phase—undissolved, crystalline COM. Moreover, the DSC thermograms indicate the presence of a single exothermic peak at around 30 °C, which can be related to the cold crystallization of COM.

Moreover, rather no effect of processing conditions on the glass transition temperature of the polymer matrix/IBU and polymer matrix/IBU/COM systems was observed. This parameter shows neither a decreasing nor an increasing trend with increasing extrusion temperature.

**Figure 6:**
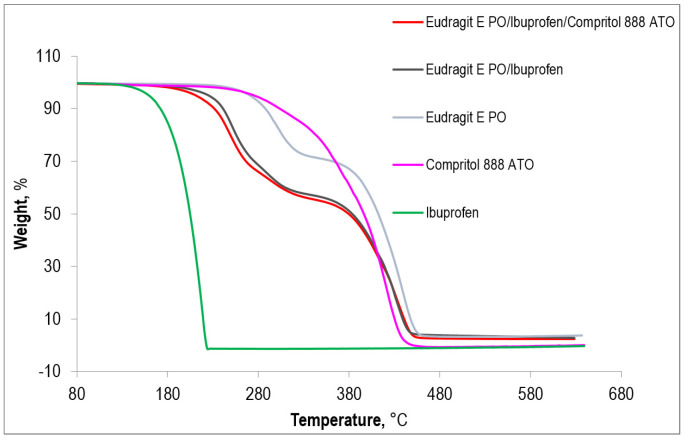
TGA curves of investigated systems: extrudate containing 75 wt% of Eudragit EPO and 25 wt% of IBU, extrudate containing 67.5 wt% of Eudragit EPO and 22.5 wt% of IBU and 10 wt% of COM, extrudate of Eudragit EPO, and additionally pure, without processing IBU and COM.

**Figure 7:**
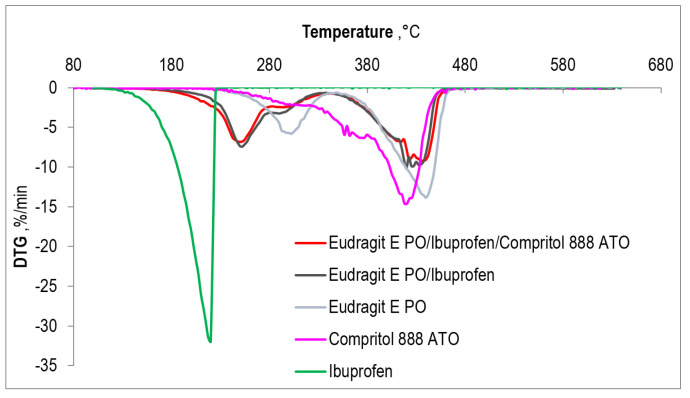
DTG curves of investigated systems: extrudate containing 75 wt% of Eudragit EPO and 25 wt% of IBU, extrudate containing 67.5 wt% of Eudragit EPO and 22.5 wt% of IBU and 10 wt% of COM, extrudate of Eudragit EPO, and additionally pure, without processing IBU and COM.

A comparison of the thermogravimetric curves obtained for three different compositions: Eudragit EPO, the Eudragit EPO/ibuprofen system, and the Eudragit EPO/ibuprofen/plasticizer system with the TGA curves of pure ibuprofen and Compritol 888 ATO is shown in Figure 6. The thermogravimetric curves of the extrudates extruded at different processing temperatures are quite the same. Moreover, Figure 7 shows the DTG curves for all the above-mentioned samples.

The TGA curves indicate complete decomposition of ibuprofen and Compritol 888 ATO, which proceeds with the formation of gaseous products only, which differs from the decomposition of the matrix and resulting mixtures, where approximately 4% of the residual mass remains in the final stage of the process. It can therefore be concluded that Eudragit EPO is clearly responsible for the presence of coke after combustion of the sample.

The TGA and DTG curves show that the compound with the lowest thermal stability is ibuprofen. These data will confirm the information contained in Table 3, which shows the temperatures corresponding to 1%, 2%, 3%, 5%, and 10% weight loss of the composition. It was assumed that sample decomposition begins when 2% of the mass is lost. In the case of ibuprofen, such a loss appears already at a temperature of 141.7 °C, which is more than 100 °C lower than the two percent decomposition temperature of Eudragit EPO. This indicates the highest sensitivity of ibuprofen to temperature among all tested materials. Both on the thermogravimetric curve and its first derivative, it can be seen that ibuprofen undergoes a clear one-stage decomposition, the maximum rate of which falls at the temperature of 220 °C.

The polymer matrix, Eudragit EPO, undergoes two-stage decomposition and the maximum rate of the first degradation step occurs at about 300 °C and the second at 440 °C. The decomposition of Compritol 888 ATO can be described as multi-stage, but the decomposition peaks overlap, which makes it impossible to determine them precisely. The last stage of decomposition is clearly visible, with the maximum rate at 418 °C. The matrix/drug and matrix/drug/plasticizer systems have a slightly more complicated distribution, which can be described as three-stage, with the third stage of decomposition probably being complex, or the image obtained may result from the appearance of noise on the thermogram. In a binary and ternary system, the first compound to decompose is ibuprofen. However, the degradation of Compritol 888 ATO and Eudragit EPO probably also begins in the same temperature range, as indicated by a small peak appearing around 290 °C.

The temperature at which both of these systems assume their maximum rate of decomposition is around 417 °C. The introduction of ibuprofen into the polymer matrix contributed to the acceleration of Eudragit EPO decomposition. The maximum rate of the first stage of polymer matrix decomposition occurs at a temperature of 300 °C and of the second stage at 437.5 °C. These temperatures decrease successively to 245 °C and 420 °C after adding ibuprofen. On the other hand, the introduction of the active substance into the multimolecular compound resulted in an improvement in the thermal degradation characteristics of ibuprofen—a delay in its decomposition. The maximum rate of decomposition of the pure compound occurs at 220 °C, while the rate of decomposition contained in the matrix is at 245 °C. The effect of Compritol 888 ATO on the resistance of the matrix/drug system is insignificant; however, it manifests itself in a slight shift of the composition’s disintegration towards lower temperatures. As for the plasticizer itself, it decreased its thermal stability after introducing into a matrix made of Eudragit EPO.

#### 3.1.2. XRPD Analysis

**Figure 8:**
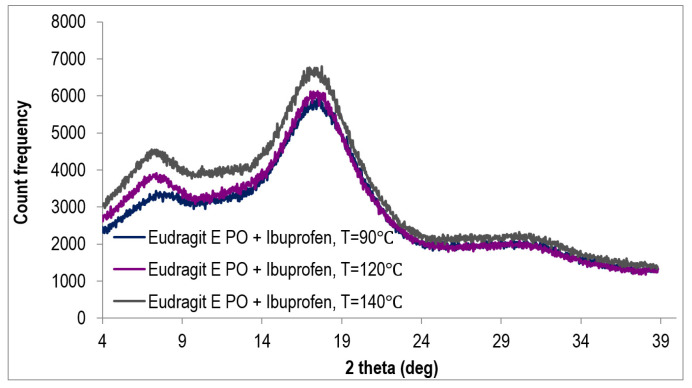
Diffractogram of Eudragit EPO/IBU systems extruded in various processing conditions (90 °C, 120 °C, and140 °C).

**Figure 9:**
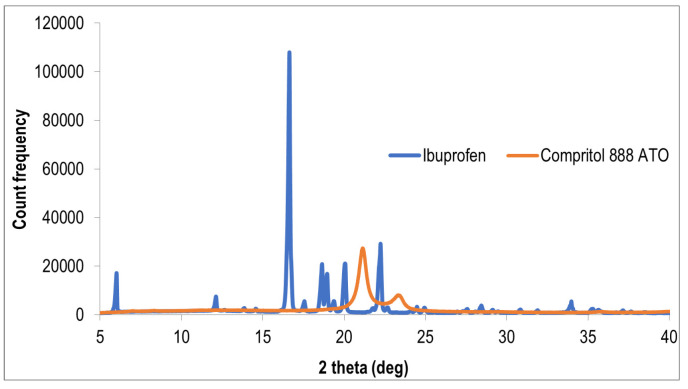
Diffractograms of pristine ibuprofen and Compritol 888 ATO.

The diffractograms recorded for three Eudragit EPO/IBU extrudates obtained in different temperatures of the processing (90 °C, 120 °C, and 140 °C), are shown in Figure 8. Additionally, diffractograms of pristine IBU and pristine COM, are presented at Figure 9. The presented diffractograms of extrudates show three broad amorphous bands, the location of which is consistent with literature reports presenting a diffraction pattern characteristic of the amorphous structure of Eudragit EPO polymer [23]. The agreement of the peak positions indicates the amorphous structure of all three mixtures of the polymer with the active ingredient IBU. There are no ibuprofen peaks visible, as can be seen from the comparison with the diffractogram obtained for pristine IBU (Figure 9). This fact proves the complete dissolution of ibuprofen in the polymer matrix and the presence of API in amorphous form.

**Figure 10:**
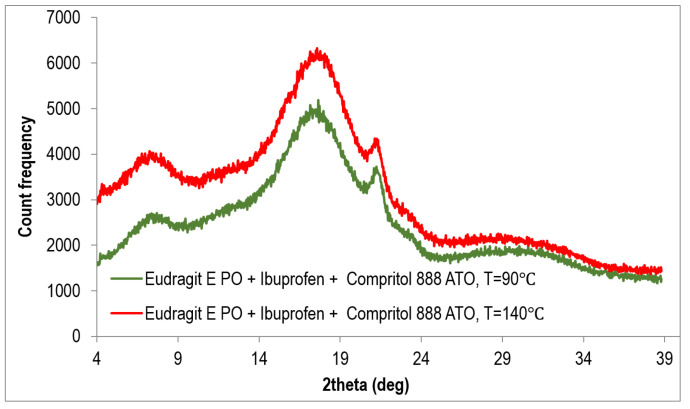
Diffractogram of Eudragit EPO/IBU/COM systems extruded in various processing conditions (90 °C and 140 °C).

Two extrudates consisting of a mixture of polymer, IBU, and plasticizer COM were also subjected to XRPD testing (Figure 10). One additional peak can be seen in the diffractograms in relation to the diffractograms obtained for the polymer matrix/API systems without the addition of a plasticizer (Figure 8). This peak is associated with the presence of a crystalline substance, and its position corresponds to the angle 2θ of 21°. According to the diffractogram of Compritol 8888 ATO presented at Figure 9 and information provided in the article [24], the crystal form of Compritol 888 ATO gives a signal in the form of two peaks—the first, more intense, at 2θ equal to 21.22° and the second, less intense, at 2θ equal to 23.43°. These data confirm that the peak present in the obtained diffraction patterns comes from Compritol 888 ATO, which is at least partially in the crystalline state. The presence of only one peak may result from the fact that the plasticizer content in investigated systems is low, i.e., 10% by weight.

The obtained results are consistent with those obtained by the DSC method and confirm the amorphous nature of Eudragit EPO/IBU extrudates and the presence of a small crystalline phase of the plasticizer in Eudragit EPO/IBU/COM extrudates.

#### 3.1.3. Intermolecular Interactions

**Figure 11:**
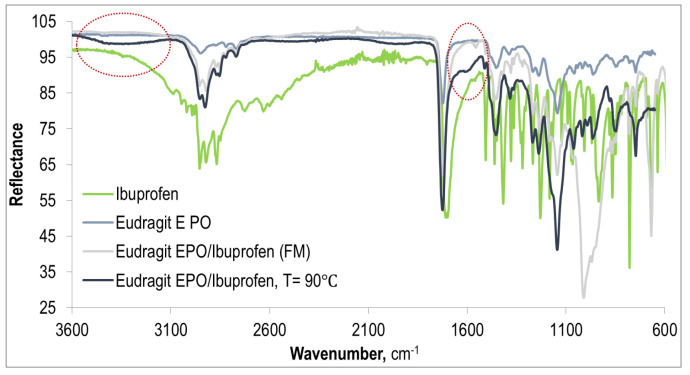
FTIR-ATR spectra of pure Eudragit EPO and ibuprofen as well as the matrix/drug system extruded at 90 °C.

Figure 11 shows a comparison of the FTIR-ATR spectra recorded for pure Eudragit EPO and pure ibuprofen as well as the spectrum obtained for a physical mixture of Eudragit EPO with IBU as well as one of the prepared extrudates (E-EPO/IBU, T = 90 °C). A comparison of the spectra obtained for the physical mixture and the extrudate of E-EPO/IBU shows differences that may indicate the presence of interactions between the polymer and API in the extrudate. This is indicated by a wide absorption band at wave numbers in the range of 3600 cm^−1^–3200 cm^−1^, which appeared on the spectrum of the extrudate of Eudragit EPO/IBU. It can be related to stretching vibrations of the N–H group, resulting from the protonation of the tertiary amino group of Eudragit EPO. This would then indicate a reaction between ibuprofen as an acid and the amino group of the polymer matrix. This band may also be superimposed by a band of moisture-related vibrations. Additional confirmation of intermolecular interactions between components of the extrudate is a slight peak appearing at the wavenumber of 1590 cm^−1^, which is not observed in the spectrum of the physical E-EPO/IBU mixture. This band corresponds to the vibrations of the carboxylate anion and suggests the formation of a IBU salt with the Eudragit EPO in the case of extrudate.

**Figure 12:**
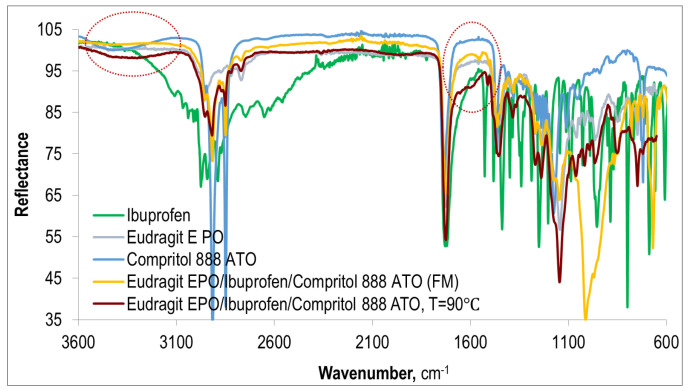
FTIR-ATR spectra of pure Eudragit EPO and ibuprofen as well as the Eudragit EPO/IBU/COM system extruded at 90 °C.

Also, in the FTIR spectrum of the Eudragit EPO/IBU/COM system, which is presented in Figure 12 together with the spectra of a physical mixture of E-EPO/IBU/COM and pure Eudragit EPO and pure ibuprofen, absorption bands indicating interactions between the components of the composition are visible. Despite the overlapping of the absorption band of the hydroxyl group of Compritol 888 ATO with the absorption band of stretching vibrations of the N–H group, resulting from the protonation of the tertiary amine group of Eudragit EPO, the interactions between ibuprofen and the polymer matrix are still confirmed by the peak at the wavenumber equal to 1590 cm^−1^. This proves that the plasticizer has no effect on the process of salt formation between the active compound and the polymer matrix.

The FTIR spectra of all Eudragit EPO/IBU and Eudragit EPO/IBU/COM systems analyzed in this work, extruded in different technological conditions, do not show significant differences. Absorption bands in most cases coincide; only their intensity changes slightly. However, no correlation was found between the size of the peaks corresponding to the content of the formed salt (absorption peak at 1590 cm^−1^) and the processing temperature. Thus, it can be concluded that the technological conditions used during the extrusion process probably do not have a significant impact on the material obtained.

Confirmation of the interactions between the components of the composition may positively affect the stability of the obtained solid polymer/IBU dispersions. Thus, stability tests were carried out in accelerated conditions, i.e., at 40 °C and 75% humidity for a period of 28 days and 3 months. DSC thermograms and XRPD diffractograms taken after preparation of the tablets and after the aging period were compared, and no changes were observed. Thus, stable amorphous solid dispersions were obtained.

### 3.2. Tablets Preparation

Prepared tablets containing extrudates with and without Compritol were tested, and results are presented in Table 4. Tablets with Compritol are slightly harder and more brittle and have a much longer disintegration time than tablets without the addition of Compritol. In addition, the uniformity of the composition is greater in the case of tablets without the addition of Compritol. Thus, Compritol slightly deteriorates the properties of the prepared tablets.

### 3.3. Dissolution Tests

**Figure 13:**
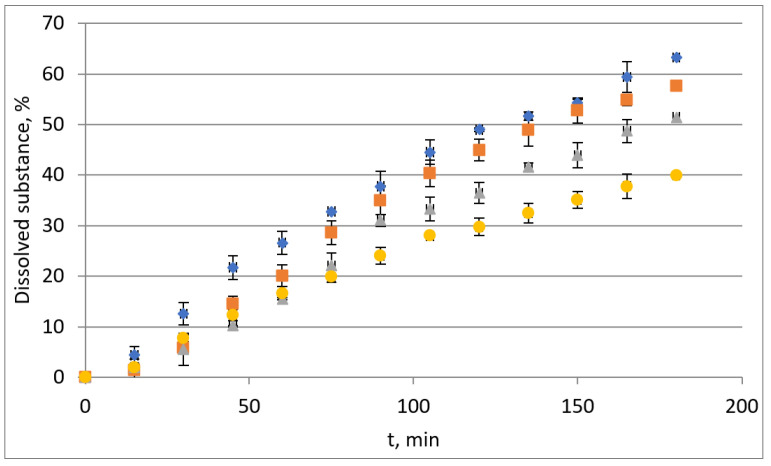
Ibuprofen release profiles from tablets containing Eudragit/IBU or Eudragit/IBU/COM extrudates obtained at a processing temperature of 90 °C or 140 °C in artificial gastric juice (pH 3.1) and: yellow circle—E-EPO 75%/IBU 25% + 10% COM extruded at 90 °C, gray triangle—E-EPO 75%/IBU 25% + 10% COM extruded at 140 °C, orange square—E-EPO 75%/IBU 25% extruded at 90 °C, blue diamond—E-EPO 75%/IBU 25% extruded at 140 °C.

Tablets, containing the obtained extrudates at the processing temperature of 90 °C and 140 °C, prepared in accordance with the described methodology, were subjected to the study of the release of the active substance in the gastric juice solution. Two types of extrudates were investigated, i.e., with and without of the addition of plasticizer Compritol 888 ATO. Obtained results are presented on Figure 13.

Both the processing temperature and the addition of a COM affect the release of IBU from the tested tablets. In the gastric juice, solution for which the E-EPO tablets are intended, the best release profile was obtained for tablets with the Eudragit EPO/IBU extrudate obtained at 140 °C without the addition of a plasticizer. Within 180 min 63.3% of the IBU was released from the tablet. On the other hand, from the tablet containing the extrudate obtained at a lower processing temperature, i.e., 90 °C, 57.7% of IBU was released. Significantly lower values of the released active substance were recorded in the case of tablets containing E-EPO/IBU/COM extrudates, with only 40% of IBU released in the case of the extrudate obtained at a lower processing temperature (90 °C) and 51.5% of IBU when processing temperature was 140 °C. Thus, the addition of a COM, which improves the processing of the polymer matrix/API system, has a delaying effect on the release of the API. It is possible that this will be a modified release, but unfortunately, longer release studies have not yet been conducted, which are in further research plans.

Since the release process is characterized by better parameters when it contains the extrudate obtained at 140 °C, and the other parameters of the extrudates obtained at different processing temperatures are the same, the best extrusion temperature for studied system Eudragit EPO/IBU will be 140 °C.

## 4. Conclusions

We have successfully obtained amorphous solid polymer/API dispersions using Eudragit EPO as the polymer matrix and 25 wt% ibuprofen as the API. We used different extrusion temperatures from 90 °C to 140 °C determined on the basis of DSC phase transition measurements and TGA thermal stability measurements of ingredients used in investigations. Obtaining of amorphous solid dispersions of polymer/API was confirmed by both DSC and XRPD studies. They were stable in accelerated conditions (40 °C, 75% RH) for studied period of time, i.e., three months. In the case of using the Compritol 888 ATO plasticizer as a compound to improve the extrusion parameters, it turned out that part of the plasticizer does not mix with the polymer/API system and remains in the crystalline phase. However, it improved processing parameters. IBU release profiles from tablets showed that tablets containing extrudate without plasticizer obtained at 140 °C had the best properties.

## Figures and Tables

**Table 1:** Temperatures of individual zones of the barrel.

Die Zone	Zone 8 °C	Zone 7 °C	Zone 6 °C	Zone 5 °C	Zone 4 °C	Zone 3 °C	Zone 2 °C
**90**	90	80	70	70	70	70	40
**100**	100	90	90	90	90	70	40
**120**	120	110	110	110	110	70	40
**140**	140	130	120	120	120	70	40

**Table 2:** List of all tablet ingredients for the tableting process of the third and fourth formulation.

Without Compritol 888ATO			With Compritol 888ATO		
Ingredient	Amount		Ingredient	Amount	
	g/tab.	%/tab.		g/tab.	%/tab.
**Extrudate: polymer+API**	0.2	38.230	**Extrudate:** **polymer + API + plasticizer**	0.22	40.516
**Lactose monohydrate**	0.1	19.120	**Lactose monohydrate**	0.1	18.416
**Corn starch**	0.005	0.960	**Corn starch**	0.005	0.921
**Calcium hydrogen phosphate dihydrate**	0.05	9.560	**Calcium hydrogen phosphate dihydrate**	0.05	9.208
**Microcrystalline cellulose**	0.15025	28.730	**Microcrystalline cellulose**	0.15025	27.670
**Sodium carboxymethyl starch**	0.0125	2.390	**Sodium carboxymethyl starch**	0.0125	2.302
**Colloidal silica**	0.00025	0.050	**Colloidal silica**	0.00025	0.046
**Magnesium stearate**	0.005	0.960	**Magnesium stearate**	0.005	0.921
**Total amount**	**0.523**	**100**	**Total amount**	**0.543**	**100**

**Table 3:** Weight loss temperatures of 1%, 2%, 3%, 5%, and 10% of active ingredient, matrix, matrix/drug, and matrix/drug/plasticizer.

	Weight Loss, %	1	2	3	5	10
Sample						
		**Temperature, °C**				
Ibuprofen		126.5	141.7	149.5	159.1	171.7
Eudragit EPO		222.6	247.8	258.3	270.9	287.7
Compritol 888ATO		143.0	234.2	253.4	276.9	303.1
Eudragit EPO + IBU		159.7	194.4	208.8	224.7	241.0
Eudragit EPO + IBU + COM		149.2	183.5	196.8	211.6	231.8

**Table 4:** Test results of tablets containing prepared extrudates E-EPO/IBU with or without COM.

TEST		Tablet EPO/IBU		Tablet EPO/IBU/COM	
Initial		Extrudate, 90 °C	Extrudate, 140 °C	Extrudate, 90 °C	Extrudate, 140 °C
**Appearance**	Visual	Round, white to off-white tablets		Round, white to off-white tablets	
**Average mass**	Ph. Eur. 2.9.5.	Limit 0.497–0.549 mg (±5%)		Limit 0.516–0.570 mg (±5%)	
		0.501–0.528 mg	0.503–0.531 mg	0.521–0.539 mg	0.518–0.552 mg
**Hardness**	Ph. Eur. 2.9.8.	90–115 N	87–120 N	84–134 N	95–129 N
**Disintegration time**	Ph. Eur.	Limit until 15 min			
		Mean 1′32″	Mean 0′57″	Mean 12′09″	Mean 8′46″
**Fragility**	Ph. Eur. 2.9.7.	Limit <1.0%			
		Weight loss: 0.5%	Weight loss: 0.2 %	Weight loss: 0.6 %	Weight loss: 0.4%
**Content uniformity**	Pf. Eur. 2.9.6.	AV: L1 (n = 10): ≤15.0 AV: L2 (n = 30): ≤25.0 Limit 85–115% 42.50–57.50 mg			
		Min 99.7% Max 98.4%	Min 102.1% Max 95.0%	Min 85.6% Max 96.8%	Min 95.0% Max 97.1%

## Data Availability

The data presented in this study are available upon request from the corresponding author.
